# Treatment of lumbar degenerative disc disease-associated radicular pain with culture-expanded autologous mesenchymal stem cells: a pilot study on safety and efficacy

**DOI:** 10.1186/s12967-017-1300-y

**Published:** 2017-09-22

**Authors:** Christopher Centeno, Jason Markle, Ehren Dodson, Ian Stemper, Christopher J. Williams, Matthew Hyzy, Thomas Ichim, Michael Freeman

**Affiliations:** 1 Centeno-Schultz Clinic, Broomfield, CO 80021 USA; 2 Regenerative Sciences, LLC, 403 Summit Blvd Suite 201, Broomfield, CO 80021 USA; 3 Immune Advisors, LLC, San Diego, CA USA; 4 CAPHRI School of Public Health and Primary Care, Maastricht University, Maastricht, Netherlands

**Keywords:** Degenerative disc disease, DDD, Culture-expanded stem cells, Mesenchymal stem cells, MSC, Radicular pain, Autologous, Intradiscal injection, Bone marrow, Regenerative medicine

## Abstract

**Background:**

Degenerative disc disease (DDD) is a common cause of lower back pain with radicular symptoms and has a significant socioeconomic impact given the associated disability. Limited effective conservative therapeutic options result in many turning to surgical alternatives for management, which vary in the rate of success and also carry an increased risk of morbidity and mortality associated with the procedures. Several animal based studies and a few human pilot studies have demonstrated safety and suggest efficacy in the treatment of DDD with mesenchymal stem cells (MSCs). The use of bone marrow-derived MSCs for the treatment of DDD is promising and in the present study we report on the safety and efficacy findings from a registry based proof of concept study using a percutaneous intradiscal injection of cultured MSCs for the management of DDD with associated radicular symptoms.

**Methods:**

Thirty-three patients with lower back pain and disc degeneration with a posterior disc bulge diagnosed on magnetic resonance imaging (MRI) met the inclusion criteria and were treated with culture-expanded, autologous, bone marrow-derived MSCs. Prospective registry data was obtained at multiple time intervals up to 6 years post-treatment. Collected outcomes included numeric pain score (NPS), a modified single assessment numeric evaluation (SANE) rating, functional rating index (FRI), measurement of the intervertebral disc posterior dimension, and adverse events.

**Results:**

Three patients reported pain related to procedure that resolved. There were no serious adverse events (i.e. death, infection, or tumor) associated with the procedure. NPS change scores relative to baseline were significant at 3, 36, 48, 60, and 72 months post-treatment. The average modified SANE ratings showed a mean improvement of 60% at 3 years post-treatment. FRI post-treatment change score averages exceeded the minimal clinically important difference at all time points except 12 months. Twenty of the patients treated underwent post-treatment MRI and 85% had a reduction in disc bulge size, with an average reduction size of 23% post-treatment.

**Conclusions:**

Patients treated with autologous cultured MSCs for lower back pain with radicular symptoms in the setting of DDD reported minor adverse events and significant improvements in pain, function, and overall subjective improvement through 6 years of follow-up.

NCT03011398. A Clinical Registry of Orthobiologics Procedures. https://clinicaltrials.gov/ct2/show/NCT03011398?term=orthobiologics&rank=1

## Background

Low back pain (LBP) is one of the most common medical complaints worldwide and the most prevalent cause of disability among Americans between 45 and 65 years of age [1]. LBP is the second most frequent reason for a physician office visit, and the third most common diagnosis associated with surgical procedures [2]. Approximately 70–85% of adults will experience LBP at some point during their lifetime [3, 4]. The high incidence of LBP also comes with high medical costs, resulting in the highest economic burden among all musculoskeletal complaints [5].

The cause of LBP can be difficult to pinpoint, as there are multiple potential anatomic sources of pain in the lumbar spine. Pain can result from degenerative and traumatic changes in the intervertebral disc, facet joint degeneration, deep soft tissues surrounding the spine (e.g. myofascial pain or multifidus muscle atrophy), and intraabdominal/retroperitoneal pathology (i.e. cholecystitis or kidney stones) [6, 7]. A predominant source of LBP is the intervertebral disc (IVD); it has been estimated that as much as 40% of all LBP is attributable to derangement of the IVD [8]. The rate of degenerative disc disease (DDD), which is a normal consequence of aging and typically begins in the 3rd decade of life, is influenced by vascular supply, genetics, biomechanics, occult infection, and direct trauma [9–11]. Disc degeneration is a unidirectional and irreversible process; currently there are no existing conservative or surgical treatment options to slow or reverse the process. Progressive degeneration of the IVD alters the integrity of the cartilaginous annulus fibrosis that surrounds the nucleus pulposus. As the annulus begins to lose resiliency, it can develop tears, which can allow the nucleus to migrate posteriorly and cause the disc to bulge. The nucleus can also herniate through the posterior annulus, a pain-sensitive structure which is innervated by the sinuvertebral nerve [12]. Chemical irritants from the nucleus can exude from the disc and cause a painful inflammatory response in the spinal nerve root, resulting in radicular symptoms (i.e. pain or weakness in a dermatomal pattern). Alternately, herniated nuclear and annular tissue can exert direct mechanical pressure on the spinal nerves, causing the same type of radicular symptoms [13].

Most episodes of LBP are relatively mild and self-limited, and easily managed with conservative therapies (i.e. physical therapy, chiropractic care, massage, acupuncture, medications, ice/heat, etc.). A subgroup of LBP patients who fail conservative measures undergo minimally invasive procedures (i.e. epidural steroid injections, nerve blocks, radiofrequency neurotomy or ablation, etc.) or ultimately have low back surgery (i.e. lumbar fusion, discectomy, or laminectomy). The rate of spinal surgery for the treatment of symptomatic DDD has increased dramatically in recent decades; low back surgery rates increased 220% from 1990 to 2001 and doubled again from 2000 to 2009 [14, 15]. The efficacy of surgery for LBP is variably reported in the literature, with some studies demonstrating no improvement in outcomes when compared with conservative treatments [16–19]. Complication rates associated with spine surgery are not insubstantial; deep vein thrombosis, infection, and myocardial infarcts occur in 6.6% of initial surgeries and in 6.3% of revisions [20, 21].

A minimally invasive therapy with regenerative or degeneration mitigating capabilities is an ideal evolution of current therapies. In a 2003 publication using a rabbit model, Sakai et al. demonstrated that transplantation of culture-expanded autologous mesenchymal stem cells (MSCs) into the nucleus of degenerated discs slowed the degenerative process and improved annular integrity, providing proof of the concept [22]. While subsequent small clinical studies of the human patients have shown favorable results and good safety [23–26], no larger scale or long term studies have been published as of 2016.

Another possible therapy for the DDD and associated radicular pain is the use of platelets which contain many key anabolic growth factors [27]. For example, platelet derived growth factor have also been shown to increase peripheral nerve health by optimizing Schwann cell re-myelination [28]. In addition, a recent clinical trial demonstrated that platelet rich plasma mitigated pain and increased function when injected into discs with early DDD [29].

In the present study we describe the analysis of prospectively gathered data over a 7 year period from a treatment registry of consecutive patients presenting with LBP and diagnosed with a posterior disc bulge and radicular pain, who were treated with an intradiscal injection of autologous culture-expanded MSCs with platelet lysate. We describe the outcomes over time in terms of patient symptom severity and function, as well as in pre- and post-treatment MRI findings. This present study is an extension of a previously published case series of five patients receiving intradiscal culture expanded mesenchymal stem cells [30].

## Methods

### Study design and clinical protocol

Consecutive patients presented to a single interventional pain practice for evaluation with complaints of low back pain from May 2008 to June 2015. An estimated 1833 patients entered a patient registry for tracking outcomes after receiving an interventional percutaneous injection for spinal treatment. Of these patients, 53 had received an intradiscal injection of autologous culture-expanded MSCs. A chart review was conducted using the following inclusion/exclusion criteria narrowing down our sample to 33 patients.

### Inclusion criteria used for treatment of intradiscal injection of autologous culture-expanded MSCs

1. DDD with a posterior disc bulge confirmed on MRI that was consistent with history and exam.
2. Clinical diagnosis of radicular pain.
3. Failed conservative treatment (i.e. physical therapy, modalities, and multiple medications).
4. Failed interventional therapy (i.e. epidural steroid injections at the suspected level of the pain generator).
5. Unwillingness to pursue surgical option.

### Exclusion criteria

1. Active non-corrected endocrine disorder potentially associated with symptoms (i.e. hypothyroidism, diabetes).
2. Active neurologic disorder potentially associated with symptoms (i.e. peripheral neuropathy, multiple sclerosis).
3. Severe cardiac disease.
4. Pulmonary disease requiring medication usage.
5. History of active neoplasm within the past 5 years.
6. Anemia.
7. Prior therapeutic intradiscal injection (i.e. hyaluronic acid or Fibrin).

The treatment protocol was approved by an Institutional Review Board (HHS OHRP #IRB00002637). All patients were required to undergo an informed consent process and sign an informed consent form before entering the study. Patients were enrolled into a treatment registry and prospectively followed using an electronic system, ClinCapture software (Clinovo Clinical Data Solutions, Sunnyvale, California) that generates an automated post-treatment questionnaire for evaluation at 1, 3, 6, 12, 18, and 24 months after receiving treatment, and annually thereafter. The registry collected three patient reported outcomes in addition to adverse events:

1. *Percent improvement* This metric ranged from −100% worsened to 100% improved, with pre-treatment baseline at 0%, and was normalized to the modified single assessment numeric evaluation (SANE) rating via flooring the percent improvement scores at 0%; therefore all subpopulations reporting −100 to 0% improvement were normalized to 0%, indicating no improvement.
2. *Pain score* The numeric pain score (NPS) is a 0–10 scale where 0 = no pain and 10 = worst possible pain).
3. *Function* The functional rating index (FRI) is a 0–100 scale with 0 being functionally independent with no disability and 100 being severely disabled.

Patients were asked to complete the NPS and FRI before the procedure to obtain a baseline score for these measures. A more detailed protocol for the registry data collection methods has been previously published [31–34].

The modified SANE metric was selected based on clinical experience and felt to be a summary of the meaningfulness of overall clinical result [35]. Clinically, the NPS is a reliable and valid metric of pain that gives an objective quantifiable value that has been extensively studied in patients with chronic LBP [36]. The FRI is a 10 question survey asking patients to rate their level of pain and ability to engage in various activities (i.e. walking, traveling, standing, recreation, etc.) [27]. The FRI has been shown to be a reliable and valid measure of pain and disability of the spinal musculoskeletal system in a clinical setting [30].

### Isolation and expansion of MSCs

A detailed description of the isolation and expansion technique has been previously described by the authors [33, 34]. A brief summary of the procedure is described below.

One week prior to the bone marrow aspiration procedure, patients were given instructions to discontinue use of corticosteroids and/or nonsteroidal anti-inflammatory drugs. On the same day as the bone marrow aspiration, 20–60 cc of heparinized venous blood was collected and used to create platelet lysate (PL); yielding high concentrations of PDGFs, to be used for the pre-injection as described in the injection protocol below. The PL was prepared by centrifuging the blood at 200*g* for 6 min to separate platelet rich plasma (PRP) from the red blood cells (RBCs). PRP was drawn off and stored at −20 °C to produce pellet platelet bodies. The platelet bodies were re-centrifuged at 1000*g* for 6 min and the resulting supernatant was drawn off to produce the PL. A 10–20% solution of PL with minimum essential medium alpha (A-MEM) was prepared for cell culture media.

Each patient was placed in the prone position on an operating room table with the target area prepped and draped in a sterile fashion. Using C-arm fluoroscopy for accuracy, the bilateral posterior superior iliac spine (PSIS) was anesthetized at 1–3 locations using 1% lidocaine (Hospira, Inc.-NDC 0409-4276-02). A sterile disposable trocar was used to manually enter the marrow cavity at each location, harvesting 10 cc of bone marrow aspirate from each site into a 30 cc syringe containing 30,000 units of Heparin (Aurobinda pharma limited-NDC 63739-964-25). Approximately 30 cc of bone marrow aspirate (BMA) was harvested from the left PSIS and the procedure was repeated at the right PSIS for a total of 60 cc of BMA drawn per patient. The BMA was then transferred to a clean room laboratory for culturing.

For the culturing process, the BMA was centrifuged at 200*g* for 4–6 min to separate nucleated cells from the RBCs. The nucleated cells were then pelleted by centrifuging at 1000*g* for 6 min. The pellet was washed once in a phosphate buffered solution (PBS), the cells were counted, and then re-suspended in culture medium. After 6–12 days in hypoxic culture (i.e. 5% oxygen) MSC colonies that developed were harvested by using an animal origin-free trypsin-like enzyme (TrypLE Select-Gibco, 12563). For the expansion of MSCs, cells were re-plated at a density of 6000–12,000 cells/cm^2^ in A-MEM with 10–20% PL, 5 μg/mL doxycycline, and 2 IU/mL of heparin, and grown to approximately 80% confluence. Passage 0 consisted of primary cells derived from the nucleated cell population, and each subsequent subculture of MSCs was considered one further passage. After 2–5 passages, MSCs were harvested, washed, and suspended in 20% PL with PBS in readiness for the injection.

### IVD injection protocol

*Pre-injection* 2 weeks prior to autologous cultured cells re-injection, each patient presented to the clinic for pre-injection. Approximately 20 cc of heparinized venous whole blood was drawn and processed as described above to produce the PL injectate. Pre-injection consisted of a transforaminal epidural using 3–5 cc of PL at the level of the intended target. Patients were placed prone on the operating table with sterile preparation, and a transforaminal epidural using 3–5 cc of PL as injectate under fluoroscopic guidance was completed.

**Fig. 1:**
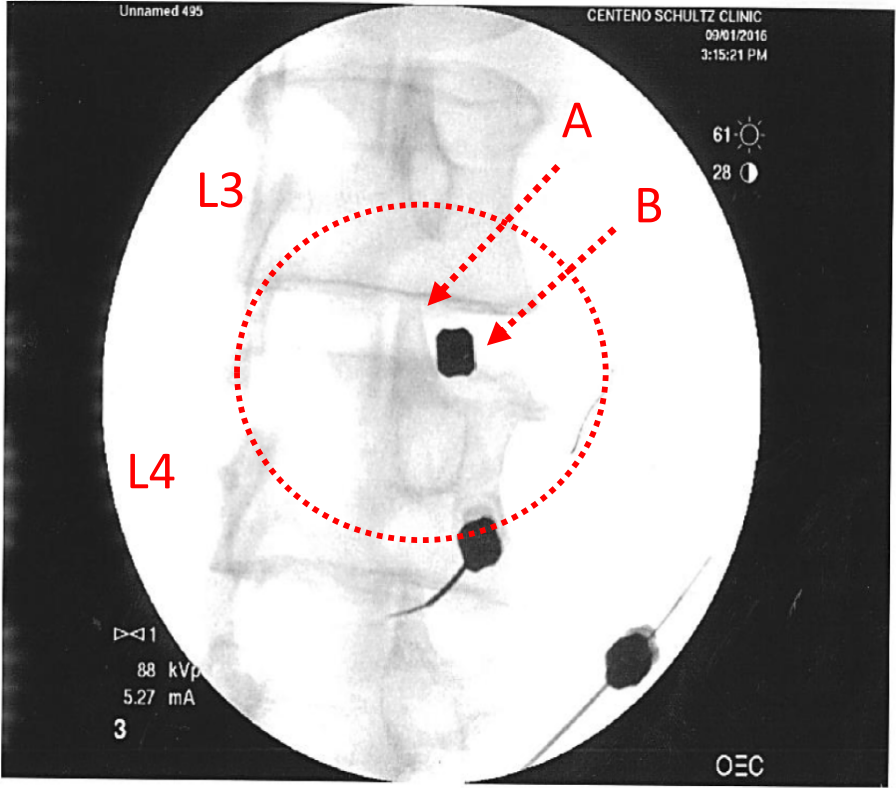
Intradiscal approach targeting the L3-L4 IVD (circled area). A = superior articular process; B = needle

*Re-injection* Once MSCs had been sub-cultured and suspended in 10–20% PL, they were prepared for re-injection. A small sample of the prepared MSCs was karyotyped and if any abnormalities were identified the procedure was cancelled. Patients were brought into the procedure room and placed in a prone position on operating room table, and after a standardized sterile preparation, the injection sites were anesthetized using 1% lidocaine (Hospira, Inc.-NDC 0409-4276-02). Prior to MSC injection, contrast (Omnipaque 300 mg/mL-NDC 0407-1413-51; diluted 1:1 or 1:2 with PBS) was injected to ensure proper targeting of the injectate. Under fluoroscopic guidance, cultured MSCs were percutaneously injected into the symptomatic disc(s) via standard discography access over the superior articular process with the starting point being as lateral as possible to allow as much of the injectate as possible to be injected into the posterior disc annulus (Fig. 1). MSCs were injected with autologous PL 10–20%. Approximately 1–3 mL of cultured MSCs was injected into the symptomatic lumbar disc. Patients were prescribed pain medicine to be used as needed for 1–14 days and placed on restrictions as tolerated. Physical therapy post-treatment was not controlled for but was encouraged.

*Post-injection* 2 weeks after the IVD re-injection of MSCs, each patient presented to clinic for a subsequent injection. This consisted of a transforaminal epidural using 3–5 cc of PL at the level of the intended target. Approximately 20 cc of heparinized venous whole blood was drawn and processed as described above. With patients placed in the prone position with sterile preparation, a transforaminal epidural using 3–5 cc of PL as the injectate was completed using fluoroscopic guidance.

A non-treating physician collected all MRI data using RadiAnt DICOM (Version 1.9.16.7446, 64-bit) viewer software. The pre-treatment and last dated surveillance MRI T2 weighted sagittal images of the injected level were matched as closely as possible for comparison. To quantify posterior IVD dimension the measurement tool in the Radiant viewer software was utilized. A vertical line was used to measure the perpendicular distance between the most posterior edge of the corresponding vertebral bodies to the most posterior point of the posterior disc in millimeters [37–39]. The pre- and post-treatment posterior IVD dimensions could thus be objectively compared.

### Statistical analysis

Patient demographic information (age, gender and body mass index (BMI)) and modified SANE scores were described using the mean and standard deviation (SD). Baseline self-reported scores (NPS and FRI) were described similarly. Two-sample dependent t-tests were used to analyze the differences between baseline and post-treatment scores for the FRI, NPS, and IVD dimension change. Patients without baseline scores were excluded from the respective analyses. All analyses were performed utilizing R, a software environment for statistical computing and graphics, version 3.2.2. Figures were created using R, version 3.2.2 [40].

## Results

### Demographics

There were 33 patients fitting the inclusion criteria who were followed in the treatment registry between May 2008 and September 2015. Twenty-one (63.6%) patients were males and 12 (36.4%) were females with a mean age of 40.3 years (SD = 14.2; min = 19; max = 72). BMI was calculated for 24 of the patients and averaged 24.8 (SD = 2.8; min = 20.83; max = 33.9). All patients were treated with a single level IVD injection, with L5-S1 level treated most commonly (22/33; 66.67%), followed by L4-L5 IVD. Of the 33 patients, 4 patients had the same disc injected multiple times, with 3 patients having 3 injections. These 3 patients were excluded from the post-treatment analysis. The average cell dose was 2.3 × 10^7^ (range 1.73 × 10^6^ to 4.5 × 10^7^). No differences or trends were noted in outcomes related to cell dose.

### Safety

Of the 33 patients who received treatment, 9 reported a complaint in the complications tracking system as part of the ongoing registry follow-up. Of those 9 patients, 3 (9%) were adjudicated by the attending physician as an adverse event (AE) related to the procedure. All 3 of the reported adverse events were pain related and resolved, as no patient reported an AE at multiple time points. No serious adverse events such as death, paralysis or neurologic deficit were reported. Two patients (6%) underwent a later spine surgery. There were no cases of infection (discitis) upon follow up. One AE was reported as a large herniated nucleus pulposus (HNP), which occurred months after the injection. This complication could have been related to the trauma from the needle procedure, or could have simply been a progression of the degenerative process [33].

### Modified SANE rating

**Fig. 2:**
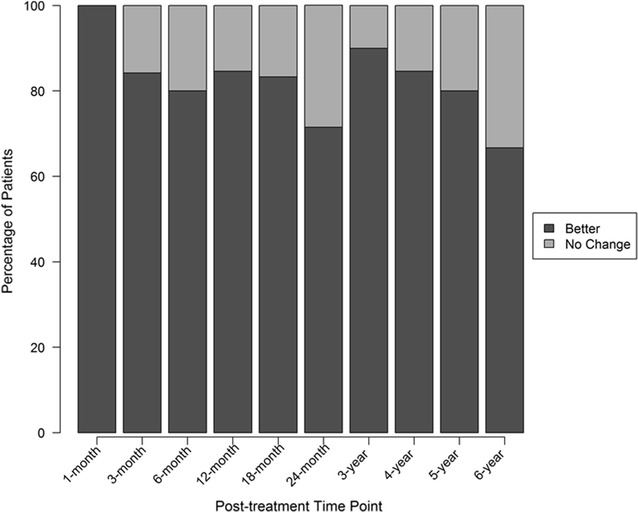
Patient-reported improvement. Percent of patients reporting improvement categorized as better (modified SANE > 0% improvement) or no change (modified SANE = 0% improvement) at each post-treatment time point up to 6 years. Number of patients reporting at each time: 1 month (N = 5); 3 month (N = 19); 6 month (N = 15); 12 month (N = 13); 18 month (N = 12); 24 month (N = 7); 3 years (N = 10); 4 years (N = 13); 5 years (N = 10); 6 years (N = 9)

Thirty of the 33 patients responded with self-reported modified SANE ratings after receiving treatment (three patients lost to follow-up). The overall average for the last reported SANE rating was 48.2%, recorded at an average of 40.6 months post-treatment with 50.4% reporting greater than or equal to 50% improvement. At 3-years post-treatment, 90% of patients reported > 0% improvement. Detailed modified SANE data is characterized in Table 1 and displayed in Fig. 2.

**Table 1:** Outcome questionnaire data

	Pre-treatment	1-month	3-month	6-month	12-month	18-month	24-month	3-year	4-year	5-year	6-year
Mean SANE rating		42	47	51	45	51	43	60	57	56	53
Mean NPS change score	5.2	1.5	1.6**	1.4	0.6	2	1.2	2*	2.5**	3.7**	3.3*
Mean FRI change score	61	19	20**	17	4.9	17	17	12	19	35**	30

Outcome questionnaire data for baseline and all post-treatment time points up to 6 years. Includes scores for modified SANE rating (post-treatment only), numeric pain score (NPS), and functional rating index (FRI). Statistical difference from baseline mean: * p < 0.1, ** p < 0.05

### Numeric pain scores

Of the 33 patients, 25 (76%) reported baseline data, and those without baseline data were excluded from the change score analysis. Average baseline NPS was 5.2. Post-treatment average NPS ranged from 3.3 to 3.6 between months 1–24 and from 1.9 to 2.3 between 2 and 6 years after receiving treatment. To determine improvement in pain, we calculated a change score by subtracting the post-treatment from the baseline score. The average last reported change score for NPS across all post-treatment time points was 2.3 at an average of 38.9 months. Numeric pain change scores were found to be significant at 3 months, 4 years, and 5 years (p < 0.05). See Table 1 for the breakdown of NPS data.

### Functional rating index

Baseline FRI data was provided by 16 (48%) of the 33 patients whose scores averaged 60.5 before receiving treatment. Post-treatment average FRI scores ranged from 31.1 to 44.9. Change score averages showed improvements in FRI scores ranging from 4.9 at 12 months (N = 6) to 35 at 5 years (N = 4). The average change score across last reported post-treatment time points was 17.6 at 36.7 months. FRI change in scores was significantly different than baseline at 3 months and 5 years post- treatment (p < 0.05). The minimal clinically important difference (MCID) for FRI is 8.4 points [41]. The average change in scores exceeded the MCID at all but the 12-month time point. Full FRI details are shown in Table 1.

### Posterior IVD dimension

**Fig. 3:**
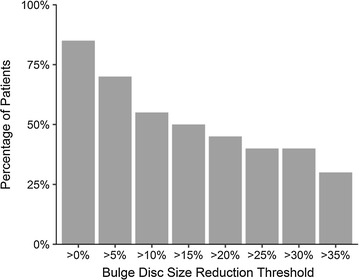
Post-treatment reduction in disc bulge size by percentage threshold (N = 20). Patients at each threshold have obtained a reduction in disc bulge size of more than the percentage shown. For example, 85% of patients demonstrated a reduction of greater than 0%

**Fig. 4:**
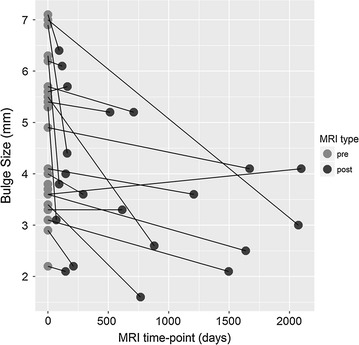
IVD herniation bulge measurements on MRI at pre-treatment compared to last post-treatment MRI shown by number of days after treatment. Lower values at the post-treatment time point indicates reduction in disc bulge size

**Fig. 5:**
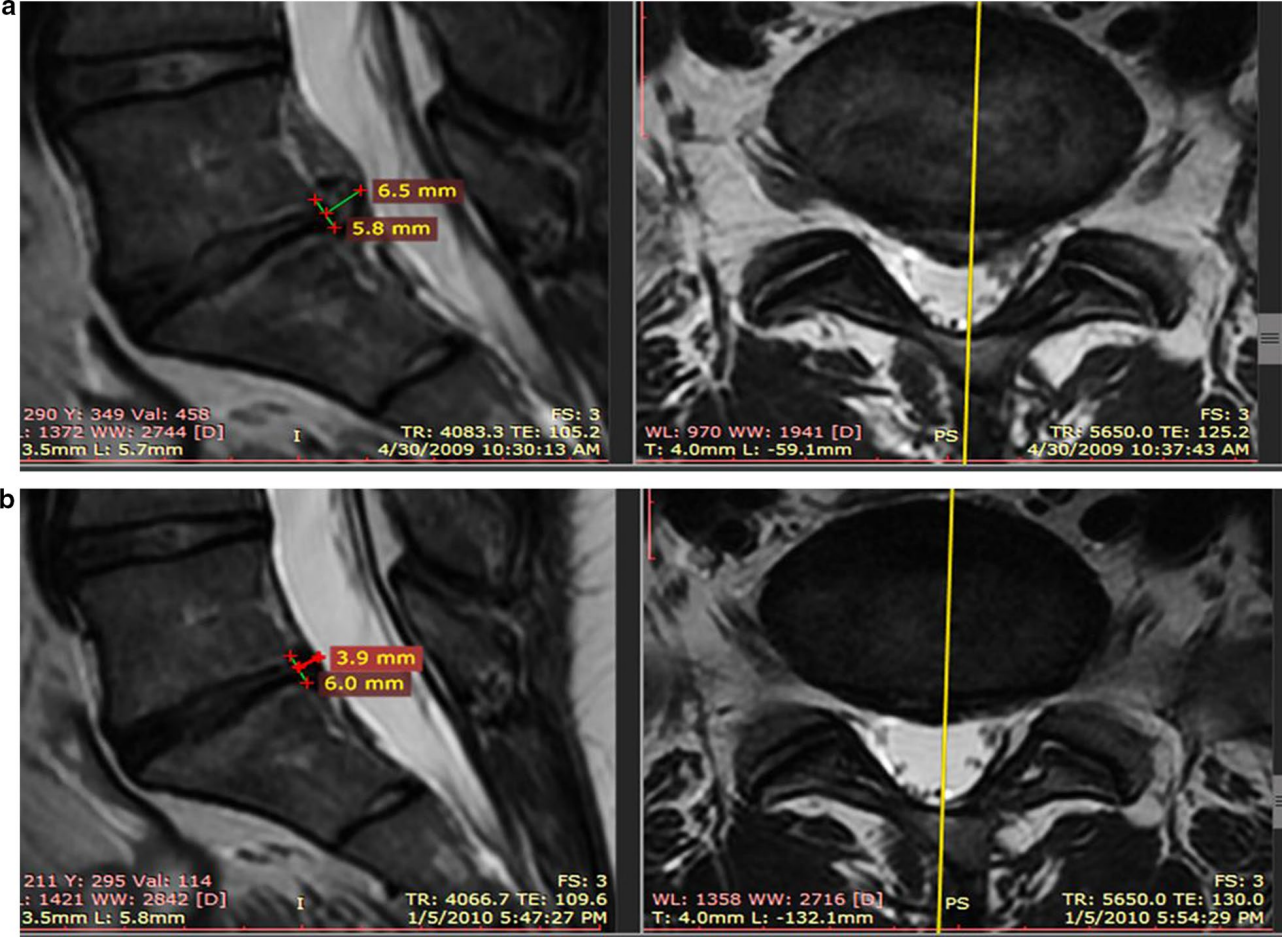
Comparison of disc bulge size on MRI. **a** Pre-treatment MRI, L5-S1, 4-30-2009; 6.5 mm posterior disc bulge. **b** 5 months post-treatment MRI, L5-S1, 1-5-2010; 3.9 mm posterior disc bulge

After the re-injection, 20 of the 33 patients underwent post-treatment MRI scans. Of the 20 patients, 17 (85%) displayed a decrease in posterior disc bulge dimension. The percentage of patients achieving various posterior IVD dimension reduction is shown in Fig. 3. On average, there was a 23% reduction of IVD projection beyond the posterior intervertebral margin at an average of 6 months post-treatment. See Fig. 4 for the change of posterior disc projection reduction in millimeters by the post-treatment MRI time point. See Fig. 5 for an example of pre- and post-MRI measurements.

A secondary analysis stratified the 20 patients into 2 different groups based on bulge size change in order to examine correlation with symptoms and function; (i.e. bulge size change ≥ 25% and bulge size change < 25%). Eight of the 20 patients had ≥ 25% reduction in bulge size. For the ≥ 25% group: average disc bulge size change was 42.9%. For the less than 25% group: average disc bulge size change is 6.3%. Patients in the group with ≥ 25% change in disc bulge size reported significantly lower average NPS score at 6 months (p < 0.05) post-treatment compared to the group of patients with a bulge size change < 25%. There were no statistically significant differences between the two different groups on any of the other outcome questionnaires.

## Discussion

In the present study, we report the largest case series to date for the treatment of DDD with intradiscal autologous mesenchymal stem cells. We aimed to examine the efficacy of an injection of autologous culture-expanded MSCs into the intervertebral disc and annulus of DDD patients with radicular symptoms and a posterior disc bulge. For up to 6 years after receiving treatment, patients are self-reporting continued clinically significant improvements, decreased pain and increased function through our prospective registry.

In addition to patient-reported outcomes, changes in IVD posterior projection or bulge beyond the vertebral body were also measured. A decrease in posterior disc bulge was detected in 85% of patients at an average of 6 months post-treatment. In determining how much of a decrease in bulge size measurement is clinically significant, we found that those patients with at least a ≥ 25% reduction in disc bulge reported significantly lower pain scores at 6 months compared to patients with a < 25% change in bulge size. No further differences were found for the other outcome measures based on this 25% reduction in bulge size stratification. This is not surprising, given that many studies have shown a disconnect between imaging findings on lumbar MRI and pain [42]. Therefore, our study is the first known to the authors, where we saw reduction in bulge size correlating with outcome metrics. Furthermore, the < 25% change group may not of had enough regression of posterior bulge to create a statistically significant functional outcome on patient reported outcome scores.

Development of DDD involves complex cellular and molecular interactions that may be directly and indirectly related to both hereditary factors and impaired nutrient supply [43]. The IVD constitutes the largest avascular organ in the human body having 80% of its nutrients supplied via diffusion from end plate capillaries of the adjacent vertebral bodies, which are known to atrophy throughout one’s life [43]. Limited vascular supply creates a unique microenvironment within the IVD, characterized by a low oxygen environment with high acidity compared to blood plasma pH [44, 45]. The environment created in DDD results in limited repair capability that is demonstrated in the reduction of nucleus pulposus cells. Limited cellular content leads to changes in the extracellular matrix (ECM), such as decreased proteoglycans and type I and type II collagen, which are responsible for the integrity and stability of the IVD [10, 46, 47]. The progressive structural decline also has an impact on the function of the IVD, inhibiting its natural ability to distribute forces properly. These alterations in biomechanics result in annular tears that progress to posterior bulges and neural compression, accounting for a significant portion of LBP.

As a result of the hypoxic nature of the disc, this intradiscal injection based treatment protocol used hypoxic culture conditions to replicate the environment of the degenerating disc. While a few prior small case series (N = 10) [25] have published on the effects of both bone marrow concentrate and culture expanded bone marrow derived MSCs, none have reported on the use of this treatment protocol to reduce disc bulge size [32–34]. For example, while Orozco and colleagues reported 10 patients treated with normal oxygen environment culture-expanded bone marrow-derived MSCs for the treatment of chronic low back pain caused by DDD with encouraging outcomes, only some improvement in DDD MRI metrics were reported [48]. They reported no changes in IVD height, but they did note significant improvement with IVD hydration based on MRI analysis. In similar fashion, we have seen improvement in morphology of IVD with an improvement in posterior disc bulge size. While we did not quantitatively measure IVD hydration, qualitatively we did not note consistent improvements in this disc property.

Given the multi factorial cascade of DDD and multiple structures involved; researchers have published on the theoretical effects of MSCs in delaying, arresting, and possibly reversing the DDD cascade [49]. One possible mechanism for this effect would be stabilizing or reversing the degenerative changes seen in the annulus fibrosis. The annulus fibrosis is composed of fibroblasts that create type 1 and type 2-collagen, proteoglycans, elastin, and other proteins [50]. This amalgamation of dense protein structures allows the annulus to withstand the gravitational and mechanical forces translated through the vertebral bodies. Disruption of the posterior annulus may be seen on MRI imaging as a high intensity zone (HIZ) which may develop into a disc protrusion [51, 52]. Furthermore, The HIZ has been shown to harbor increased levels of tumor necrosis factor alpha [53]. MSCs have multiple functions that can account for the improvements seen in intervertebral disc morphology. Our hypothesis was that MSCs will differentiate into fibroblasts which will deposit new collagen and extra-cellular matrix; therefore reinforce the dense protein amalgamation of the annulus fibrosis, preventing further posterior herniation, and decreasing the chemical inflammation contributing to chemical radiculitis. One explanation for our results may be changes in collagen density and decreased pro inflammatory catabolic cytokines.

The use of autologous cultured MSCs for intervertebral disc pathology does have potential risks including those normally attributed to and beyond the known risks involved with intradiscal injection procedures. Rubio et al. highlighted the importance of biosafety with expansion of MSCs ex vivo as long-term culture (4–5 months) may increase the risk of spontaneous transformation; however, this pivotal study was later retracted because the authors were unable to replicate their results [54, 55]. Moreover, several additional studies have not identified any malignant transformation with prolonged culturing [56–59]. In the current study, potential risks for chromosomal abnormalities or tumorigenic transformation were reduced by only using short-term culture (less than 28 days). Samples of the expanded cells were karyotyped prior to re-injection and if abnormalities were noted, the re-injection would have been cancelled per safety protocol; however no patient in the current study had abnormal karyotyping. The re-injection procedure into the IVD poses two additional concerns (1) causing an infection or discitis and (2) needle trauma to the IVD resulting in further degeneration of the IVD [60, 61]. One reported complication (that occurred months after the injection), was a large HNP that required surgical decompression. The observed large post-treatment HNP could have been due to the trauma caused by the needle entry or perhaps via excessive NP cell proliferation or ECM overproduction and was reported as part of our 2011 safety paper [33]. Outside of this possible complication, the registry data analysis revealed only a few adverse events reported by patients that were determined to be related to the procedure. The majority of these were due to post-treatment pain that resolved with conservative care. No neoplasms were observed on imaging in the area of injection nor did any patient develop any new neoplastic events following the procedure.

Limitations of this study include missing data, lack of a control group or placebo group, post-treatment MRIs not conducted on everyone, possible therapeutic effect of the pre- and post-injection with platelet lysate (PL), the presence of radicular pain in addition to axial lower back pain, lack of concordant discography done prior to the intradiscal injection, lack of a standardized post-treatment rehabilitation protocol, and a small sample size. As discussed earlier, this group of patients had missing data, which is common with registry data. Although patients were contacted multiple times via email, phone and/or mail, there may be inherent bias introduced by those that do respond. However, at least one outcome data point was collected on 88% of patients for all outcome measures collected. Post-treatment MRI surveillance results were recorded in 20/33 (61%), ideally 100% of those treated would have underwent post-treatment MRI analysis to allow for a more complete post-treatment analysis of disc bulge size. Also, using the same MRI scanner for all images would minimize any confounding factors associated with the use of different MRI scanners. A placebo or other effects not related to the autologous cultured MSCs cannot be ruled out. To date, we are unaware of any published studies using PL for the treatment of axial LBP. In-vitro animal studies have shown some beneficial effects of platelets to aid in nerve regeneration but no in vivo studies have been published for the treatment of axial LBP or radicular pain [62, 63]. Interestingly, there has been reported success with the perineural injection of PRP for peripheral neuropathy treatment [64–67]. One could reason that the growth factors contained in PL could have led to improvement in the radicular component of pain and therefore overall improvement in patient reported outcomes; however, the PL epidural was unlikely to result in improvement in the size of the disc bulge that was observed in the majority of those with post-treatment MRIs. Discogenic pain is traditionally axial in nature, but when there is mechanical compression or tearing in the annulus fibrosus, concomitant radicular pain is common in addition to axial LBP. Herniation of the nucleus pulposus has been associated with the pathological process known as chemical radiculitis and occurs due to the leakage of inflammatory cytokines with resultant irritation of the adjacent nerve roots [68, 69]. The absence of pre-procedural discography for a definitive diagnosis by the reproduction of concordant axial LBP in patients with multiple levels of pathology can be viewed as a limitation. However, in patients with chronic LBP, false-positive rates can range from 40 to 80% [70, 71]. Furthermore, there is considerable controversy surrounding the associated risks with discography; including the progression of disc degeneration, new disc herniation, and discitis [72]. Given the fact that the treatment for this study was an intradiscal injection and already carried with it the previously mentioned associated risks, the authors decided to forgo adding the extra risk by not doing the pre-procedural discogram. Further studies can consider concordant discography, limit the inclusion criteria to axial LBP with seating intolerance, and possibly the use of new diagnostic biomarkers [e.g. fibronectin-aggrecan complex (FAC)] to aid in the diagnosis of discogenic LBP [73]. However, the diagnostic utility of using biomarkers from local tissue remains unclear [74]. Patients were encouraged to participate in physical therapy (PT) and variations in individual PT were not accounted for and included in results analysis. Additionally, the sample size of 33 with 20 post-treatment MRI measurements is small and limits this study’s power.

An argument can be made that disc protrusions and/or extrusions improve over time without any treatment. This was not controlled for in this current paper. In a study by Komori et al. who examined 77 patients with radiculopathy, only extruded free fragments showed a frequent decrease in size naturally, while protrusions showed little to no change on follow-up imaging at a mean duration of 150 days [42]. None of our patients presented with extruded fragments and instead all presented with stable disc protrusions that had failed extensive conservative care. In fact, the mean time that our patients attempted conservative therapy for their disc protrusion was 6.08 years (26 of the 33 study patients) prior to this procedure. Additionally, only patients with stable disc protrusions that were unresponsive or minimally responsive to epidural injection were accepted into the study. Furthermore, Komori et al. [42] showed that MRI findings lag behind the clinical improvement. This may also be contributing to our findings of increased functional gain without a precise correlation with the change in disc protrusion size on post-treatment MRI analysis.

## Conclusions

The intradiscal injection of culture expanded MSCs to treat DDD with symptomatic disc bulge produced encouraging results in this registry based pilot study with imaging analysis. Results garnered showed no safety issues, substantially reduced pain, increased function, and reduced disc bulge size in most patients. While these results are encouraging, further controlled investigations, ideally utilizing randomization and crossover are needed to validate the efficacy of this promising therapy.

## Abbreviations

AE: adverse event
A-MEM: minimum essential medium alpha
BMA: bone marrow aspirate
BMI: body mass index
DDD: degenerative disc disease
ECM: extracellular matrix
FRI: functional rating scale
HIZ: high intensity zone
HNP: herniated nucleus pulposus
IVD: intervertebral disc
LBP: low back pain
MCID: minimal clinically important difference
MSC: mesenchymal stem cell
MRI: magnetic resonance imaging
NPS: numeric pain score
OA: osteoarthritis
PBS: phosphate buffered solution
PL: platelet lysate
PRP: platelet-rich plasma
PSIS: posterior superior iliac spine
RBC: red blood cell
SANE: single assessment numeric evaluation
SD: standard deviation
TNF-α: tumor necrosis factor alpha
